# Gene transcript fusions are associated with clinical outcomes and molecular groups of meningiomas

**DOI:** 10.1007/s00401-024-02708-y

**Published:** 2024-03-20

**Authors:** Naomi Zakimi, Minh P. Nguyen, David R. Raleigh

**Affiliations:** 1 Department of Radiation Oncology, University of California San Francisco, San Francisco, CA USA; 2 Department of Neurological Surgery, University of California San Francisco, San Francisco, CA USA; 3 Department of Pathology, University of California San Francisco, San Francisco, CA USA

The discovery of molecular groups of meningiomas that are associated with distinct biological drivers, therapeutic vulnerabilities, and clinical outcomes provides a framework for redefining the classification of the most common primary intracranial tumor [2–4, 6, 14, 16, 18–20, 22, 25]. Meningiomas from the Merlin-intact molecular group with favorable clinical outcomes encode recurrent short somatic variants targeting *TRAF7, KLF4, PI3K*, *POLR2A*, or the Hedgehog pathway [2, 4, 18, 21, 22, 33, 34]. Recurrent short somatic variants in meningiomas from molecular groups with poor clinical outcomes, such as *TERT* promoter mutations or *CDKN2A/B* deletions [10, 24, 29], are rare. These data suggest that alternative genomic mechanism may contribute to the formation or progression of aggressive meningiomas.

Gene fusions form when independent DNA or RNA sequences are juxtaposed through (1) chromosome structural rearrangements such as translocations, deletions, duplications, or inversions, (2) transcription read-through of neighboring genes, or (3) pre-mRNA slicing [12]. Many gene fusions are oncogenic, and gene fusions have been reported in meningiomas [11, 32], particularly *NF2* structural rearrangements in radiation-induced meningiomas [1, 26], and *YAP1* fusions in rare pediatric meningiomas [28, 30]. Associations between gene transcript fusions and clinical outcomes across molecular groups of meningiomas are unknown.

To define the landscape of meningioma gene transcript fusions, paired-end RNA sequencing data from 302 consecutive frozen meningiomas with matched DNA methylation profiling and targeted gene expression data from The University of Hong Kong [2–4] were analyzed using Arriba (Supplementary Table 1, online resource), a highly accurate bioinformatic pipeline for fusion detection in cancer transcriptomes [8]. Single-end RNA sequencing reduces the accuracy of gene transcript fusion detection [8]. Nevertheless, 81.9% of gene transcript fusions that were identified in 2 or more meningiomas using Arriba to analyze single-end RNA sequencing data from 200 frozen meningiomas from the University of California San Francisco [2–4] were also identified in paired-end RNA sequencing data from meningiomas from The University of Hong Kong (Supplementary Table 2, online resource).

To distinguish gene transcript fusions with oncogenic potential from passenger fusion sequences, the Oncofuse bioinformatic pipeline was used to calculate the probability that meningioma gene transcript fusions were biological drivers based on features in known oncogenic fusions [27]. These analyses identified 83 gene transcript fusions that were present in an average of 20 meningiomas each (range 2–186 meningiomas/fusion) across the 302 samples with high quality paired-end RNA sequencing data from The University of Hong Kong (Fig. 1a and Supplementary Table 3, online resource). There were no gene transcript fusions in 21.5% of meningiomas (*n* = 65) (Fig. 1b). Radiation-induced meningiomas (*n* = 15, average 11 fusions/meningioma) had a higher burden of gene transcript fusions than sporadic meningiomas (*n* = 287, average 8 fusions/meningioma, *p* = 0.0322, Student’s *t* test) (Supplementary Table 3, online resource). The most common gene transcript fusions were at the protocadherin (*PCDH*) locus on chromosome 5, a degenerative genomic region with multiple transcription start sites and alternative splicing patterns that may be particularly susceptible to genomic instability (*n* = 1299 of 1701 gene transcript fusion events across 302 meningiomas, 76.3%) (Fig. 1a and Supplementary Table 3, online resource). There were no associations between *PCDH* fusions and meningioma DNA molecular group [4], CNS World Health Organization (WHO) 2021 grade [14], or gene expression risk score [2]. The second most common gene transcript fusions were translocations between prostaglandin D2 synthase (*PTGDS*) on chromosome 9 and actin genes on chromosomes 7 or 17 (*n* = 160 of 1701 gene transcript fusions events across 302 meningiomas, 9.4%) (Fig. 1a and Supplementary Table 3, online resource). *PTGDS* expression has been associated with meningioma development [9], and *PTGDS* fusions were more common in Merlin-intact meningiomas (*n* = 29 of 104 meningiomas, 27.9%) compared to Immune-enriched meningiomas (*n* = 13 of 105 meningiomas, 11.3%) or Hypermitotic meningiomas (*n* = 11 of 83 meningiomas, 13.3%, *p* = 0.0037, Fischer’s exact test). There were no associations between *PTGDS* fusions and meningioma CNS WHO 2021 grade (*p* = 0.1712) [14] or meningioma gene expression risk score (*p* = 0.3644, Fisher’s exact tests) [2]. These data suggest that *PCDH* gene transcript fusions may be a common finding in meningiomas that is not relevant to molecular characteristics, while *PTGDS* fusions may contribute to the molecular group of meningiomas with the best clinical outcomes.

**Fig. 1:**
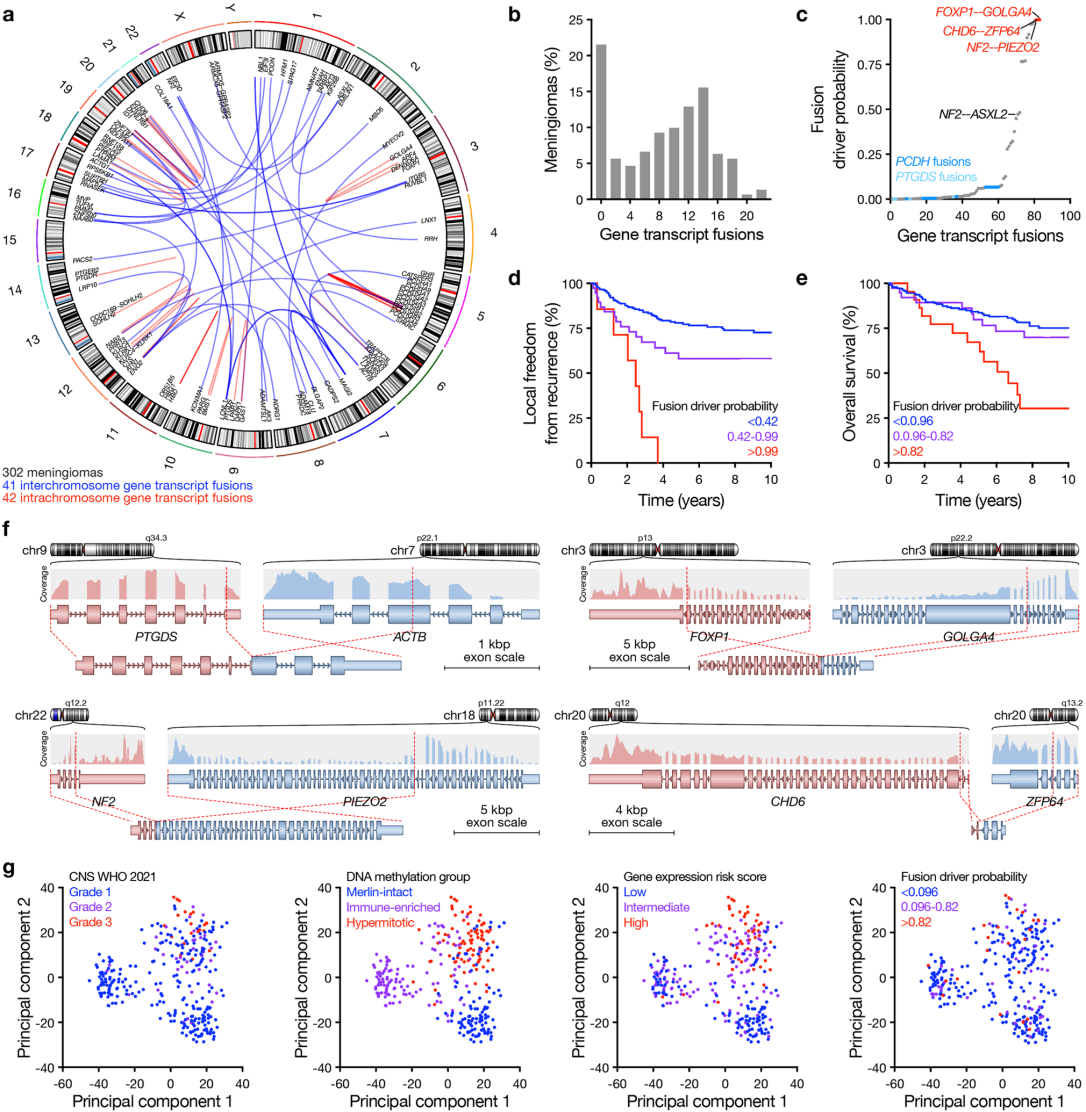
The landscape of meningioma gene transcript fusions. **a** Circos plot showing interchromosome (blue, *n* = 41) and intrachromosome (red, *n* = 42) Oncofuse results from paired-end RNA sequencing of 302 consecutive frozen meningiomas that underwent surgery. **b** Number of Oncofuse gene transcript fusions per meningioma. **c** Oncofuse driver probability of 83 gene transcript fusions across 302 consecutive meningiomas. **d** Local freedom from recurrence according to recursive partitioning analysis (RPA) of gene fusions with the highest Oncofuse driver probability per meningioma (*p* < 0.0001, Log-rank test) (see also Supplementary Fig. 1a, online resource). **e** Overall survival according to RPA of gene fusions with the highest Oncofuse driver probability per meningioma (see also Supplementary Fig. 1b, online resource) (*p* = 0.0006, Log-rank test). **f** The structure of common gene fusions (*PTGDS–ACTB*) and gene fusions with the highest Oncofuse driver probabilities (*FOXP1–GOLGA4, NF2–PIEZO2, CHD6–CFP64*) across 302 consecutive meningiomas. **g** Principal component analysis from paired-end RNA sequencing of 302 consecutive frozen meningiomas shaded by CNS WHO 2021 grade, DNA methylation group, gene expression risk score, or Oncofuse driver probability

To determine if meningioma gene transcript fusions were associated with clinical outcomes, fusion sequences were ranked by Oncofuse driver potential, which suggested that *PCDH* and *PTGDS* were low on the list of biologically-relevant fusions (Fig. 1c). Recursive partitioning analysis (RPA) was used to predict local freedom from recurrence or overall survival from the highest Oncofuse driver probability per meningioma (Supplementary Fig. 1 and Supplementary Table 4, online resource). Kaplan–Meier analysis showed that Oncofuse driver probability was significantly associated with meningioma local freedom from recurrence (Fig. 1d) and overall survival (Fig. 1e), with higher fusion driver probability associated with worse clinical outcome. The gene transcript fusions with the highest driver probabilities were novel inversions inactivating the transcription factor *FOXP1* through juxtaposition next to *GOLGA4* on chromosome 3 (*n* = 2) or the chromatin remodeler *CHD6* through juxtaposition next to *ZFP64* on chromosome 20 (*n* = 2), and novel translocations likely inactivating *NF2* on chromosome 22 through juxtaposition next to *PIEZO2* on chromosome 18 (*n* = 3) (Fig. 1a, c, f and Supplementary Table 3 and 4, online resource). A novel translocation inactivating *NF2* through juxtaposition next to *ASXL2* on chromosome 2 was also identified (*n* = 2) (Fig. 1a, c, and Supplementary Table 3, online resource). Gene transcript fusions with high driver potential were validated using annoFuse, a complementary bioinformatic pipeline for annotation and prioritization of biologically relevant fusions [7] (Supplementary Table 5, online resource). In sum, these results suggest that oncogenic gene transcript fusions are minimally conserved across meningiomas, but that bioinformatic approaches can be used to group gene transcript fusions according to their predicted biological relevance and shed light on meningioma clinical outcomes.

Principal component analysis of differentially expressed genes and annotation of CNS WHO 2021 grade [14], DNA methylation group [4], gene expression risk score [2], and gene fusion driver probability across the 302 meningiomas from The University of Hong Kong showed that meningiomas with oncogenic fusions clustered with high-grade meningiomas and molecular groups of meningiomas that are associated with poor clinical outcomes (Fig. 1g). In support of these findings, univariate Cox regression analysis showed that the highest Oncofuse driver probability per meningioma was significantly associated with local freedom from recurrence (hazard ratio 2.73, 95% confidence interval 1.48–5.04, *p* = 0.001) and overall survival (hazard ratio 1.99, 95% confidence interval 1.02–3.87, *p* = 0.042). These findings were not conserved on multivariate Cox regression analysis, where unifying polygenic molecular features such as gene expression risk score [2] remained significant but Oncofuse driver probability did not (Supplementary Table 6, online resource). There were no significant differences in meningioma gene transcript fusion burden or driver probability according to extent of resection, prior treatment, or tumor size (Supplementary Table 4, online resource).

In conclusion, these data demonstrate that gene transcript fusions are associated with clinical outcomes and molecular groups of meningiomas. Our results shed new light on genomic mechanisms that may contribute to the formation or progression of meningiomas, but it remains to be determined if gene transcript fusions can be targeted to improve treatments or clinical outcomes for patients with meningiomas. None of the gene fusions reported here or in previous publications [1, 11, 26, 28, 32], with rare exceptions [23], juxtapose kinases or other oncogenes that may be susceptible to small molecule inhibition. Thus, mechanistic interrogation of biochemical pathways that may be dysregulated in response to gene fusions, and whether such pathways are conserved in meningiomas with divergent oncogenic gene fusions, may be necessary to translate these findings into a therapeutic framework.

### Supplementary Information

Below is the link to the electronic supplementary material.

Supplementary file1 (XLSX 6862 KB)

Supplementary file2 (DOCX 219 KB)

## Data Availability

RNA sequencing, DNA methylation profiling, and targeted gene expression profiling data for all meningiomas analyzed in this study have been deposited to the NCBI Gene Expression Omnibus under accession numbers GSE183656, GSE101638, GSE212666, and GSE222054.
