# Supplementary material for: Gene transcript fusions are associated with clinical outcomes and molecular groups of meningiomas
Source: Acta Neuropathol. 2024 Mar 20;147(1):57. doi: 10.1007/s00401-024-02708-y (PMC10954959; doi:10.1007/s00401-024-02708-y)
Supplement: Supplementary file 2 — Supplementary file2 (DOCX 219 KB) [file 401_2024_2708_MOESM2_ESM.docx]

**ONLINE RESOURCES**

**
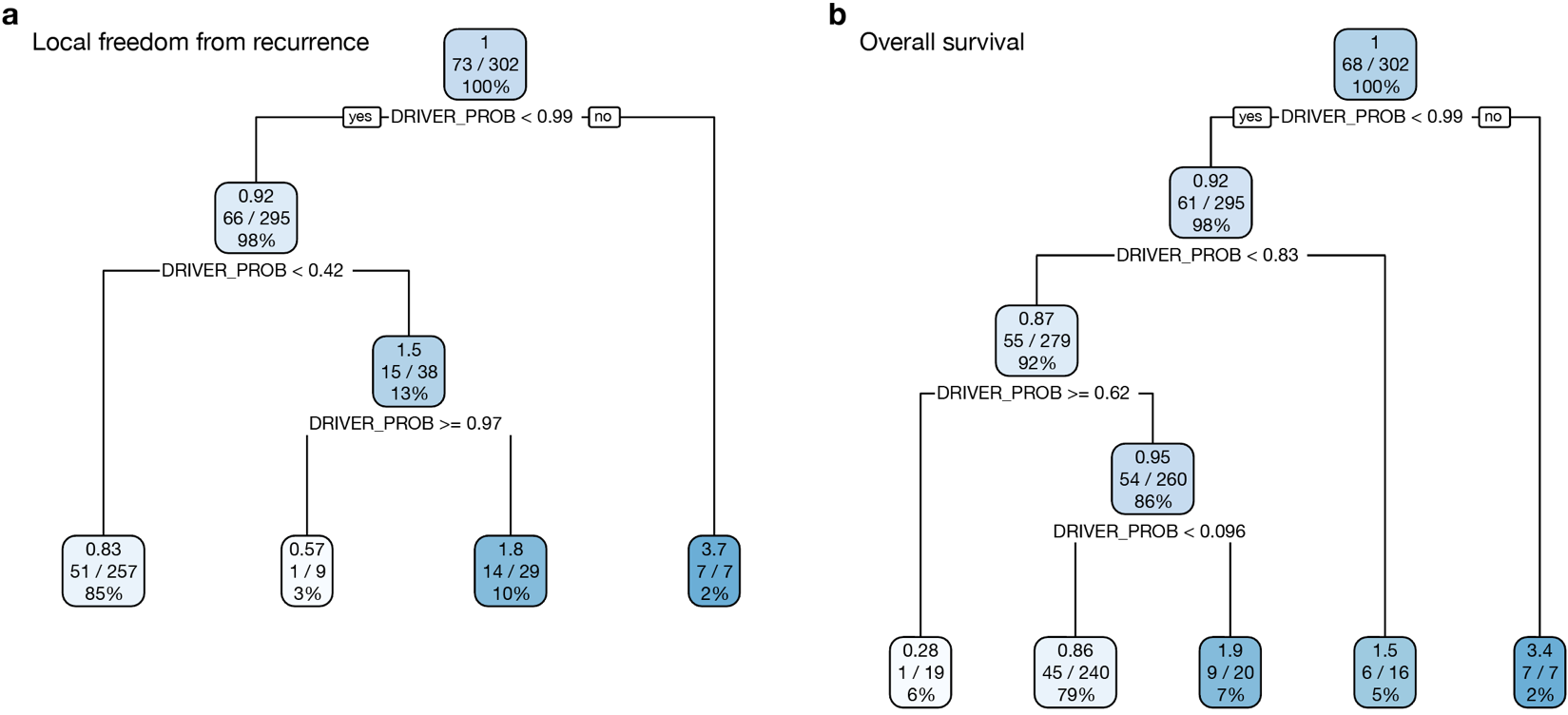
**

**Supplementary Fig. 1.** Recursive partitional analysis of meningioma gene transcript fusion driver probability to predict clinical outcomes. **a** Recursive partitioning analysis (RPA) using the highest Oncofuse driver probability per meningioma to predict local freedom from recurrence. **b** RPA using the highest Oncofuse driver probability per meningioma to predict overall survival. Meningiomas meeting cut point criteria are split left. Meningiomas not meeting cut point criteria are split right. Numerators denote number of events. Denominators denote number of meningiomas.

**Supplementary Table 1.** Arriba results.

**Supplementary Table 2.** Arriba results from UCSF validation cohort.

**Supplementary Table 3.** Oncofuse annotations.

**Supplementary Table 4.** Meningioma gene transcript fusion metadata.

**Supplementary Table 5.** Annofuse annotations.

**Supplementary Table 6.** Cox regressions.

**Methods**

The meningiomas, clinical data, nucleic acid extraction, DNA methylation profiling, and targeted gene expression profiling of the samples analyzed in this study were previously described [2–4].

*Quality assessment and trimming of sequencing reads*

The initial evaluation of raw sequencing reads was performed using FastQC v0.11.9 to assess sequencing quality. Trimming was performed using Cutadapt v3.7 to eliminate low-quality bases and Truseq adapter sequences [17]. Parameters were set to retain sequences with a minimum length of 20 bases and a quality threshold of 30. After trimming, FastQC was rerun to verify adapter removal and the quality of retained reads.

*Read alignment*

Trimmed reads were mapped against GRCh38_gencode_v37_CTAT_lib_Mar012021.plug-n-play reference genome using the STAR v2.7.10b aligner [5]. The output was prefixed with the sample’s FASTQ identifier and configured to produce BAM files sorted by coordinate. The zcat command was used for reading files, and the output included standard SAM attributes, along with unmapped reads within the main BAM file. Multi-mapping reads were limited to 50 alignments, with a minimum overlap of 10 bases for paired-end reads. Spliced alignments were required to cover at least 50% of the mate length, with a maximum of five mismatched bases allowed in the stitched junction. Additional criteria for chimeric alignments included a minimum segment length of 10 bases, output configuration for ‘WithinBAM’ and ‘HardClip’, a 10-base minimum overhang for junctions, and a 30-point maximum score drop. The minimum score separation for chimeric alignments was set to 1, with a maximum gap of 3 bases between chimeric segments and a limit of 50 multi-mapped chimeric alignments.

*Arriba gene fusion detection*

Gene fusion events were identified using Arriba version 2.4.0 with the output from STAR aligner and the GRCh38_gencode_v37_CTAT_lib_Mar012021.plug-n-play CTAT genome library build directory [31]. Arriba was executed with a stringency level set to 3. A blacklist file included in the CTAT genome library was used to filter out recurrent artifactual fusions from the analysis, improving the specificity of fusion detection.

*Gene fusion visualization*

Circos plots were generated using 3 approaches to visualize gene fusions. Gene fusions with a confidence level of “medium” or “high” identified by Arriba were visualized using the Rcircos package to differentiate between interchromosome and intrachromosomal events [35]. The UCSC HG38 human cytoband ideogram provided by Rcircos was used to provide a structural context for the data. To represent the frequency of gene fusions across samples, a frequency table was generated and linked with the data to scale the width of the connecting lines between fused genes. To validate these results, the ‘PyCircos’ package in R was used, filtering to include only fusions with a “medium” or “high” confidence level as determined by Arriba. Finally, the ‘circlize’ package in R was used, filtering to include only fusions with a “medium” or “high” confidence level as determined by Arriba.

Gene fusion visualizations were generated for individual gene fusions using Arriba v2.4.0’s built-in script draw_fusions.R to visualize gene fusions. The script created comprehensive diagrams that delineated the chromosomal locations and breakpoints of gene fusion structures involved, supporting reads, cytoband information, known protein domains affected by fusions, and Circos plots. The script created detailed plots for fusions identified with a “medium” or “high” confidence level. Annotations were provided from the GRCh38_gencode_v37_CTAT_lib reference files. Arriba was used to include Cytoband and protein domain information.

*Gene fusion annotation*

“Medium” and “high” confidence gene fusions detected by Arriba were annotated using Oncofuse v1.1.1, a framework for the prediction of the oncogenic potential of gene fusions [27]. For each sample, the input file was prepared to include the chromosome and coordinates of the fusion breakpoints. The ‘MES’ option was selected in Oncofuse to specify the mesenchymal lineage. The output included "Driver Probability Score" for each fusion. This score is a Bayesian probability estimate, with higher values indicating a higher likelihood of the fusion being oncogenic. To validate these results, “medium” and “high” confidence gene fusions detected by Arriba were annotated using annoFuse v0.92.0 [7]. FusionAnnotator v0.2.0 was used to annotate gene fusions, and annotated outputs were read and standardized using AnnoFuse. readingFrameFilter was used to include ‘in-frame’, ‘frameshift’, and ‘other’ categories, encompassing a wide range of fusion types. The artifactFilter was employed to exclude recurrent artifacts, with specific categories like ‘GTEx_Recurrent’, ‘DGD_PARALOGS’, ‘Normal’, ‘BodyMap’, and ‘ConjoinG’ being filtered out. Junction read count and spanning fragment count thresholds were set at 1 and 100, respectively. A readthrough filter was also applied to eliminate potential transcriptional readthrough events. Fusion calls were annotated using annoFuse’s reference databases, which include known oncogenes and tumor suppressors. annoFuase works by prioritizing fusions based on their previous reporting in TCGA or their involvement in known oncogenes, tumor suppressor genes, COSMIC genes, and transcription factors. This approach aids in identifying fusions most likely to be underlying oncogenic drivers in cancer. The final output included a final list of gene fusions with oncogenic potential.

*RNA sequencing principal component analysis*

Read counts were generated from STAR aligned data at the exon level using featureCounts from the Subread package v2.0.6 [13], specifying exon as the feature type and gene_id as the attribute for grouping. The following extra attributes were also included: gene_name and gene_biotype. For statistical analysis, the count data were imported into R v4.3.1 and analyzed using the DESeq2 package v1.40.2 [15]. Genes with less than 10 counts across all samples were removed. Counts were normalized and transformed using variance-stabilizing transformation (VST). For visualization and data manipulation, the following R packages were used: ggplot2, pheatmap, DESeq2, gdata, and writexl.
